# Generative Artificial Intelligence for Peer Review and Editorial Functions

**DOI:** 10.31662/jmaj.2026-0170

**Published:** 2026-06-19

**Authors:** Jaime A. Teixeira da Silva

**Affiliations:** 1 Independent researcher, Ikenobe, Kagawa, Japan

**Keywords:** generative artificial intelligence, science publishing, editorial functions, peer review

## To the Editors,

A recent editorial solidifies a growing debate on the use of generative artificial intelligence (GenAI) in the dissemination of knowledge and science, specifically in science publishing ^(1)^. This letter expands that discussion to the role of GenAI in peer review and editorial functions. Some journals and editors do not acknowledge its relevance yet in peer review, emphasizing instead human knowledge and expertise ^(2)^. However, human-centered editorial functions might not recognize the existence of GenAI-generated knowledge—for example, as GenAI-reviewed aiXiv preprints—and might inadvertently allow it to be cited in human-reviewed indexed journals ^(3)^.

Human-based peer review currently lies at the heart of science’s knowledge production, but it has an exploitative facet, especially when human peer reviewers are not remunerated. Therefore, GenAI could serve as a “peer” reviewer—potentially at a low cost—to resolve the current supply-demand imbalance, although GenAI’s biases need to be considered ^(4)^ before it is used to offer advice on research, deeming it suitable for publication. There are already cracks in the robustness of the system’s verification mechanism: the undeclared use of GenAI by authors for editing and writing or the presence of GenAI-derived text in submitted articles due to the lack of robust detection methods available to peer reviewers and editors. This suggests that the academic publishing infrastructure is not sufficiently robust or prepared to accommodate GenAI in editorial roles or as “peer” reviewers.

Indirect evidence of the unregulated use of GenAI, accompanied by the editorial inability to detect its undeclared use at the time of submission, comes in the form of postpublication retractions. In response to many letters to the editor generated by GenAI—revealing the partial failure of human peer reviewers and editors in detecting GenAI-generated text—it has been argued that such writing should remain human-centric ^(5)^, although it is important to recognize that preservation of the human status quo in peer review is becoming unrealistic. How widely is GenAI being used to replace human peer review, and are human peer reviewers employed by journals employing GenAI to complete their functions?

Academia’s split into pro-human + anti-GenAI versus pro-GenAI camps will likely emerge soon, as it may be impossible to sustainably reconcile ideological differences when faced with the threat of—or assistance by—GenAI’s infusion into the current human-centric peer review model.

## Article Information

### Author Contributions

Jaime A. Teixeira da Silva: Conceptualization, investigation, verification, writing, and editing. Generative artificial intelligence was not used for any of these functions.

### Conflicts of Interest

None

### Availability of Data and Material

Not applicable.

### Code Availability

Not applicable.
